# Wireless Ultrasound Devices in Anatomy Education: Insights from Medical Undergraduates

**DOI:** 10.1007/s40670-024-02153-2

**Published:** 2024-09-23

**Authors:** Johanna Maria de Lange, Karin J. Baatjes, Chad Marthinussen, Janine C. Correia

**Affiliations:** 1 Division of Clinical Anatomy, Department of Biomedical Sciences, Faculty of Medicine and Health Sciences, Stellenbosch University, P.O. Box 241, Cape Town, 8000 South Africa; 2 Division of Surgery, Department of Surgical Sciences, Faculty of Medicine and Health Sciences, Stellenbosch University, Cape Town, South Africa; 3 Innohealth Technologies, Stellenbosch University, Cape Town, South Africa

**Keywords:** Ultrasound; Anatomy; Medical education; Clinical application

## Abstract

Point-of-care ultrasound has become an important part of patient care, and the increased use thereof has led to a greater demand for the integration of ultrasound training in the early medical undergraduate curriculum. The use of handheld wireless ultrasound devices is not currently integrated within the undergraduate anatomy medical curriculum of Stellenbosch University and the additional value of wireless ultrasonography, in relation to the clinical practice of anatomical knowledge, therefore, warranted further investigation. This study aimed to explore undergraduate medical students’ perceptions of the use of handheld wireless ultrasound scanning to enhance knowledge and understanding of anatomy for clinical application. During the dissection sessions, students visualized anatomical structures of the musculoskeletal system, by scanning themselves, using handheld, wireless ultrasound devices. After the session, an electronic survey was distributed to the students and three ultrasonography questions were included in the routine practical test. Eighty-one survey responses were collected, with 41 of those responses being completed. The highest student agreement in the Likert scale survey was observed when assessing the convenience of practice of wireless ultrasound devices, while the lowest agreement was reported for confidence in the participant’s capability of generating ultrasound images. Two main themes were developed from the open-ended questions at the end of the survey: the instructional integration of ultrasound and ultrasound within the clinical setting. This research demonstrated that readily available access to handheld wireless ultrasound scanning has the potential to enhance students’ longitudinal learning experience and improve anatomical comprehension. As future clinicians, improved understanding could aid ultrasound application in the clinical realm.

## Introduction

Anatomy is an integral part of various medical curricula, serving as the introduction to clinical practice, with the application of living anatomy to clinical activities [1, 2, 3]. The study of anatomy involves exploring and understanding three-dimensional (3D) anatomical structures, making it an extremely visual, high-content, and practical course [4]. Medical education has traditionally utilized cadavers to teach anatomy and has been considered an integral part of the course [5]. In surgical specialties, radiology, internal medicine, dentistry, and other allied health professions, anatomy is regarded as a cornerstone of medical practice, as it directly impacts clinical care [6, 7].

Medical undergraduate students, however, perceive the subject as challenging, while having difficulty acquiring a thorough comprehension of living anatomy and applying the basic principles of medicine in clinical settings [4, 8]. Although anatomy is seen as one of the most important subjects taught in undergraduate medical programs, the contact hours dedicated to anatomy teaching are declining, while costs associated with practical dissection sessions are increasing [7, 9]. According to Elliot [10], the perception exists that gross anatomy is less important in general medical education, in comparison with other subjects, as the general structure of the human body has not changed since first recorded. The biggest concern about this potential loss of knowledge comes from senior medical professionals, who have noticed a deterioration in anatomical knowledge among medical graduates over the past decade [10]. Strengthening this observation, a study done by Lunn-Collier et al. [11], where the opinions of clinicians regarding the clinical relevance of gross human anatomy were evaluated qualitatively, showed all participating clinicians reporting that, during clinical rotations, they encountered a discrepancy between students’ anatomical understanding and its application to clinical practice. As a result, the participants commented on the benefits of having a clinically useful undergraduate medical school anatomical training, as early exposure will also ameliorate clinical judgement [11].

In ultrasonic imaging, high-frequency sound waves and reverberating echoes are used to view live image structures beneath the skin, making it an excellent visualization device in the clinical setting [12, 13, 14], with ultrafast, real-time operation [15]. Point-of-care ultrasound (POCUS), a patient-centered ultrasound examination conducted at the bedside of the patient, has become an important diagnostic tool across various medical specialties [12, 16, 17]. Portable, handheld devices or cart-based ultrasound systems can be used for POCUS. The development of cost-effective transportable ultrasound devices, which connect to smartphones or tablets through wireless technology, enables accessibility to clinicians on the front line of patient care [18, 19]. The use of handheld, wireless ultrasound devices can augment physical examinations with focused ad hoc imaging, as well as guide the choice of further investigation, in real time [20]. One of the most valuable aspects of POCUS is its ability to provide immediate visual, functional, and anatomical information at the patient’s bedside, thus aiding clinicians in the diagnostic process. Furthermore, anatomical relationships and physiology can be better understood by medical students through real-time characterization of anatomy and topographic areas [5, 20].

The increase in POCUS has led to a greater demand for the integration of ultrasound training in the early medical undergraduate curriculum, given that clinical applications of ultrasonography require clinicians to be proficient during speciality-specific needs [19]. In addition, in a clinical setting, students struggle to relate the foundational medical sciences to application in living anatomy [4]. Ultrasound has proven to be an ideal way of studying living anatomy, crosslinking foundational anatomical and physiological sciences and clinical applications [8, 12, 21, 22, 24]. Multiple studies report success in using ultrasound to enhance students’ comprehension and knowledge of anatomy [1, 2, 4, 24, 25, 26, 27], as well as strengthening teamwork among students [28].

Several factors make ultrasound an appealing tool for medical education, including its low cost, high portability, and non-invasive nature [20]. Furthermore, it does not emit ionizing radiation and has the potential to integrate basic anatomical science with clinical applications [13, 20]. The use of ultrasound to teach living anatomy enables students to observe the movement of structures during respiration and blood flow, and how structures are interconnected [5, 29].

Although ultrasound training has been progressively integrated into some medical curricula globally, a consensus has not yet been reached regarding a uniform, standardized ultrasound curriculum [2, 16, 30]. Numerous studies reported a lack of trained instructors, equipment, and training facilities, as well as too big class sizes and increased cognitive load, as major challenges in the integration of US training [2, 12, 14, 16, 21, 24, 27, 29, 31, 33, 34, 34]. In a study done by Lufler et al. [17], the financial constraint of obtaining ultrasound training machines, as well as additional faculty capacity to accommodate smaller training groups, was found to be problematic. They did, however, comment that hands-on training, in bigger student groups, will be possible when students each have their own ultrasound probe [17].

With the evolution of technology and the increase in the use of portable ultrasound within the medical and educational setting, it has been proposed that insonation could become a possible fifth pillar of bedside examination [16, 35]. As a result of the exponential growth in ultrasound usage, technological advances have led to the development of smaller, handheld devices [34]. There is frequent debate as to whether handheld devices are more efficient than cart-based systems and comparing the advantages and disadvantages of the two systems [12, 22, 35, 39].

Naganuma and Ishida [39] believe that handheld ultrasound devices will not replace cart-based ultrasound systems, due to better imaging quality, alongside the value of thorough ultrasound examinations by sonographers or radiologists. They do, however, recognize the potential of handheld ultrasound examinations. In contrast, a review done by Wilkinson and Saxhaug [35] found portable, handheld devices to be more intuitive to novice users, while their affordability, accessibility, and convenience result in employment by medical schools, to master competence prior to entering the clinical realm. Strengthening Wilkinson and Saxhaug’s [35] findings, Wang et al. [15] noted that the overall performance of ultrasound processors and transducers has been enhanced to smaller devices with higher imaging production, and decreased cost with real-time operation at ultrarapid speeds.

Advancements in medical technology resulted in the design of handheld ultrasound devices using smartphones, via wireless communication, for image capture/visualization (i.e., Bluetooth and Wi-Fi) [20, 37]. A variety of healthcare applications are increasingly utilizing smartphones with specialized attachments, due to smartphones’ enormous versatility [20, 37]. According to a study done by Bae et al. [38], healthcare professionals benefit from real-time, on-site diagnostics, with secure transmission of medical records in an industry-specific format. Furthermore, the possibility of providing patients with access to their medical records, as well as improving coordination between healthcare providers who make use of short message services, voice calls, or video conferencing, has been demonstrated. However, despite the increase in the utilization of wireless, handheld ultrasound devices, Hunt et al. [37] state that the literature should not posit claims regarding the convenience, scalability, or cost-effectiveness of these devices but use quantitative analyses to establish validity.

With the increase of ultrasonography in both the medical and educational settings, emphasis is placed on the role of this technological advancement in under-resourced areas [14, 15, 39]. Mobile communication infrastructures are now faster and more efficient at transmitting data to remote sites, providing an opportunity for diagnostic protocols in under-resourced areas [38]. In a systematic review done by Becker et al. [40], they found a significant attraction to implementing ultrasound in low- and middle-income countries, due to fewer infrastructural requirements and training sessions.

The World Health Organization stated that two-thirds of the global population lack access to medical visualization modalities [34], thus emphasizing the potential of ultrasound for better patient management and care. According to Rosman et al. [41], on average, sub-Saharan African countries have a mere 1% of the imaging modality specialists per capita compared to well-resourced areas. Furthermore, in numerous under-resourced healthcare systems, capital and human resources are so severely limited that merely providing medical care can be overwhelming, with resources for training and capacity building often unavailable [41, 42, 43].

Limited use of POCUS and emergency-point-of-care ultrasound (ePOCUS) may further be attributed to a lack of educational programs with context appropriateness pertaining specifically to disease patterns of the local communities. Other contributing factors may be limited consumable supplies, such as ultrasound gel [44, 45, 46], malfunctioning ultrasound machines, the lack of ultrasound maintenance capabilities, and the high costs associated with the purchase of portable ultrasound machines [46].

However, some of these barriers may be alleviated with the introduction of more affordable portable, handheld ultrasound systems [46]. With the development of wireless, handheld ultrasound devices, the transfer of ultrasound information and skill to rural hospitals is possible, enabling lesser-trained sonographers to obtain and explicate ultrasound images to better patient care rapidly [39]. In addition, with newly developed mobile image storage communication systems, remote locations can be securely connected to a central patient database, enabling real-time diagnosis on-site with the transfer of personal and private medical information [38, 44].

Ultrasound skills, within a South African context, are currently implemented by emergency physicians and training is mainly confined to postgraduates who are undergoing specialist training, under the regulation of the College of Emergency Medicine as part of the College of Medicine of South Africa [45, 47]. This stands in stark contrast to international literature, where ultrasound training for medical undergraduate students is viewed as non-negotiable [1, 2, 5, 8, 10, 12, 13, 14, 16, 17, 18, 20, 21, 22, 24, 24, 25, 26, 27, 28, 29, 30, 33, 34, 34, 48, 49]. Findings of a study done by Kathrada et al. [43] advocate for the incorporation of ultrasound in the undergraduate medical curriculum, as the utilization of ultrasound in clinicians’ daily practices is more likely to occur with early curriculum integration.

Handheld wireless ultrasound devices are not currently integrated into the undergraduate anatomy medical curriculum of Stellenbosch University, South Africa. The additional value of wireless ultrasonography, concerning the clinical practice of anatomical knowledge, therefore, warranted further investigation. The research question of the study was, “What are the perceptions of undergraduate medical students at the Faculty of Medicine and Health Sciences, Stellenbosch University on the use of a handheld, wireless ultrasound probe as a visualization instrument to enhance knowledge and understanding of Anatomy for clinical application?” This study aimed to explore undergraduate medical students’ perceptions of the use of handheld wireless ultrasound scanning to enhance knowledge and understanding of Anatomy for clinical application. The objectives of the study were to, based on students’ perceptions, determine if using wireless ultrasound (1) enhances knowledge and understanding of anatomical content, (2) promotes the clinical application of anatomy knowledge, and (3) could improve the healthcare system and patient outcomes, especially in under-resourced areas.

## Materials and Methods

A cross-sectional descriptive quantitative study design was followed to explore undergraduate medical students’ perceptions of the use of handheld wireless ultrasound scanning. The study was conducted at the Division of Clinical Anatomy, in the Faculty of Medicine and Health Sciences, at Stellenbosch University, and the study population included all third-year medical students (283 students) enrolled at Stellenbosch University for the 2022 academic year. The study excluded any third-year medical student, enrolled at Stellenbosch University for the 2022 academic year, who was absent during the hands-on ultrasound dissection session, as well as any other medical student year groups and postgraduate students at the Faculty of Medicine and Health Sciences at Stellenbosch University. The sampling strategy for the evaluation of the use of ultrasound in teaching and learning anatomy was that of convenience.

### Data Collection

In line with approval received from the Stellenbosch University Undergraduate Research Committee (UREC) (decision number U22/03/158) and institutional permission by the Division of Institutional Research and Planning at Stellenbosch University (IG3255), ultrasound sessions were incorporated during the routine practical dissection sessions of the musculoskeletal (MSK) system module. The anatomy of the MSK system was used as most of the anatomical structures could be visualized easily and students were able to perform the ultrasound on themselves [5, 50]. In partnership with the anatomy course coordinator, the objectives of the hands-on session were developed in advance and aligned with the anatomy dissection session outcomes.

Students were provided with an introductory session on the fundamentals of ultrasound and specific learning objectives, before scanning, which aided in orientating the students. During the introductory session, as well as the ultrasound session, a clinician and an anesthesiologist were present. The latter conducted part of the introductory session and provided basic comprehension of the principles of visualization modalities, while the clinician gave insights regarding the clinical significance of POCUS and its potential to positively impact the health system, especially in under-resourced areas. A demonstration of ultrasound examination using a U-Image™ handheld, wireless ultrasound device was also given.

The third-year medical students formed smaller groups (± 8 students) during their practical MSK sessions, and each group received a U-Image device on a rotation basis, ensuring adequate scanning opportunities for each student. The students then practiced scanning themselves, by viewing anatomical structures that form part of the MSK system, specifically the upper limb. Senior lecturers, the clinician and anaesthesiologist, as well as postgraduate students acting as demonstrators, were present during the session to observe and guide the students.

A major challenge identified in the literature is the possibility that pathology or unexpected conditions, such as pregnancy, could be detected during scans of “healthy” volunteers [29, 33, 51, 52]. It is recommended that volunteers be pre-scanned before undergoing teaching scans so that any abnormalities found can be followed up in advance [33, 52]. Due to time constraints and the size of the study population, pre-scanning of volunteers was not possible. However, the students were informed of the potential for finding existing pathology during the scan. The students who volunteered to be scanned acknowledged the possibility of abnormal ultrasound findings being revealed in front of their fellow students. These abnormalities were not detected. Should this have occurred, the clinician would have confidentially informed the volunteer(s) of the findings as soon as the ultrasound session ended and arranged for any appropriate clinical follow-up as needed [33, 52]. Moreover, the scanning of high-risk zones (such as the abdomen, thorax, and neck) [52] on students were avoided.

After the session had been completed, students were invited to complete an online survey on a password-protected web-based e-survey service, namely SUNSurveys (Checkbox Version 7.60), regarding their perceptions of handheld, wireless ultrasound probes in learning anatomy. Data collected by the usage of SUNSurveys anonymized all responses. The online survey was based on previous studies. As an add-on to the study, three ultrasonography questions were included in the routine practical test to provide insights regarding the students’ understanding of sonograms and the interpretation thereof. The three questions required identification of the structures presented on the sonograms; thus, no knowledge or comprehension of ultrasound working principles was evaluated.

### Data Analysis

Quantitative data were analyzed using descriptive statistics, which were reported as definite numbers and percentages of students. Likert scale answers from the survey were summarized by using standard deviations (SD), mean, and median Likert scale values. The responses to the surveys were summarized in five different categories for reflection: strong agreement, agreement, neutral, disagreement, and strong disagreement. Ultrasound knowledge of sonograms, which was tested during the practical routine test, was expressed as mean correct percentages out of the total amount of ultrasonographic questions within the test. All statistical analyses were computed by using computer software, namely IBM SPSS® (Version 28.0.0.0) and Microsoft Excel™ (Version 2209).

Qualitative analysis of the open-ended questions at the end of the survey was done thematically by following the method described by Creswell [53]. Coding followed an inductive approach, preventing any additional interpretation of the students’ answers, with evaluators coding students’ responses independently [17].

## Results

### Demographic Information

During the survey, demographic questions were asked of each participant, for reporting purposes. At the end of the data collection period, 81 (28.62%) survey responses had been collected, with 41 (14.49%) of those responses being completed. Of the 41 respondents, 25 (60.98%) identified as females and 16 (39.02%) as males. None of the respondents identified with the other gender categories provided in the survey. The ages of the respondents varied between 18 and 26 years.

### Quantitative Data

#### Likert Scale Survey

**Fig. 1:**
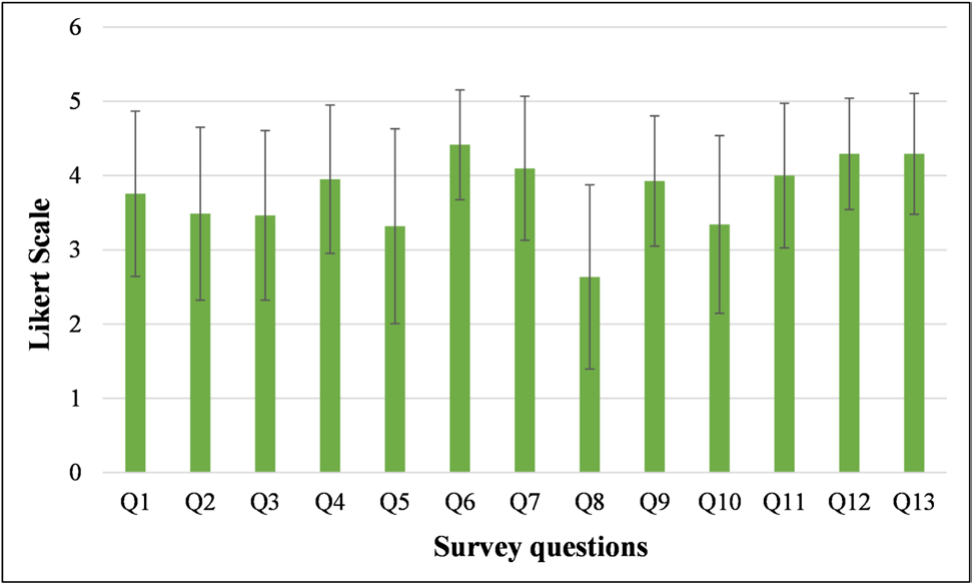
Participant responses of the Likert scale survey questions (*n* = 41). The responses are graphed as means (± SD) on a Likert scale where 1 = strongly disagree, 2 = disagree, 3 = neither agree nor disagree, 4 = agree, and 5 = strongly agree

Table 1 represents the data obtained from the Likert scale survey. All the survey questions had a high level of agreement among the participants (Fig. 1). The highest student agreement was observed when assessing the impact on the healthcare system and patient outcomes, especially in under-resourced areas, with access to wireless ultrasound devices (question 13, 4.29 ± 0.81), enhancement of longitudinal learning experiences with access to wireless ultrasound devices (question 12, 4.29 ± 0.75), and convenience for practice (question 6, 4.41 ± 0.74). The lowest agreement was reported for confidence gained with ultrasound training during the anatomical practical session for future clinical practice (question 10, 3.34 ± 1.20), the adequacy of the in-person introductory session on the fundamentals prior to the hands-on session (question 5, 3.32 ± 1.31), and confidence in the capability of generating ultrasound images (question 8, 2.63 ± 1.24).

**Table 1:** Survey questions, answer choices, and responses from third-year medical students, where 1 = strongly disagree, 2 = disagree, 3 = neither agree nor disagree, 4 = agree and 5 = strongly agree

Survey questions	Answer choice	Number of responses, *n* (%)	Median	Mean response (SD)
1. Being able to use a handheld, wireless ultrasound probe has enhanced my ability to perform ultrasound scans.	5	12 (29.3)	4	3.76 (1.11)
	4	14 (34.1)		
	3	10 (24.4)		
	2	3 (7.3)		
	1	2 (4.9)		
2. My ability to determine the meaning of ultrasound images was improved after having a handheld, wireless ultrasound probe at my disposal.	5	6 (14.6)	4	3.49 (1.16)
	4	20 (48.8)		
	3	7 (17.1)		
	2	4 (9.8)		
	1	4 (9.8)		
3. Wireless ultrasound scanning reinforced my understanding of anatomical structures.	5	6 (14.6)	4	3.46 (1.14)
	4	18 (43.9)		
	3	10 (24.4)		
	2	3 (7.3)		
	1	4 (9.8)		
4. I found the use of wireless ultrasound to study anatomy in the living human body beneficial.	5	13 (31.7)	4	3.95 (1.00)
	4	18 (43.9)		
	3	6 (14.6)		
	2	3 (7.3)		
	1	1 (2.4)		
5. The in-person introductory-explanation on the fundamentals was adequate prior to the hands-on session.	5	7 (17.1)	4	3.32 (1.31)
	4	16 (39.0)		
	3	7 (17.1)		
	2	5 (12.2)		
	1	6 (14.6)		
6. The wireless handheld ultrasonography probe is convenient for practice.	5	23 (56.1)	5	4.41 (0.74)
	4	12 (29.3)		
	3	6 (14.6)		
	2	0		
	1	0		
7. The software application for visualization modalities is convenient for usage.	5	17 (41.5)	4	4.10 (0.97)
	4	14 (34.1)		
	3	8 (19.5)		
	2	1 (2.4)		
	1	1 (2.4)		
8. I am confident in my capability to generate an image of an organ or other structures in the human body using a handheld wireless ultrasound probe.	5	4 (9.8)	2	2.63 (1.24)
	4	6 (14.6)		
	3	10 (24.4)		
	2	13 (31.7)		
	1	8 (19.5)		
9. The application of wireless ultrasound in anatomy reinforces clinical correlations.	5	8 (19.5)	4	3.93 (0.88)
	4	26 (63.4)		
	3	5 (12.2)		
	2	0		
	1	2 (4.9)		
10. Ultrasound training during the anatomy practical session gave me more confidence for future clinical practice.	5	7 (17.1)	3	3.34 (1.20)
	4	13 (31.7)		
	3	12 (29.3)		
	2	5 (12.2)		
	1	4 (9.8)		
11. Ultrasound demonstration is a necessary add-on to traditional dissection sessions during anatomy practicals.	5	14 (34.1)	4	4.00 (0.97)
	4	17 (41.5)		
	3	7 (17.1)		
	2	2 (4.9)		
	1	1 (2.4)		
12. My longitudinal learning experience at Stellenbosch University would be enhanced if I had access to a wireless ultrasound device.	5	19 (46.3)	4	4.29 (0.75)
	4	15 (36.6)		
	3	7 (17.1)		
	2	0		
	1	0		
13. The healthcare system and patient outcomes, especially in under-resourced areas, will be improved if each medical student had their own wireless ultrasound device.	5	20 (48.8)	4	4.29 (0.81)
	4	14 (34.1)		
	3	6 (14.6)		
	2	1 (2.4)		
	1	0

#### Sonogram Questions

**Fig. 2:**
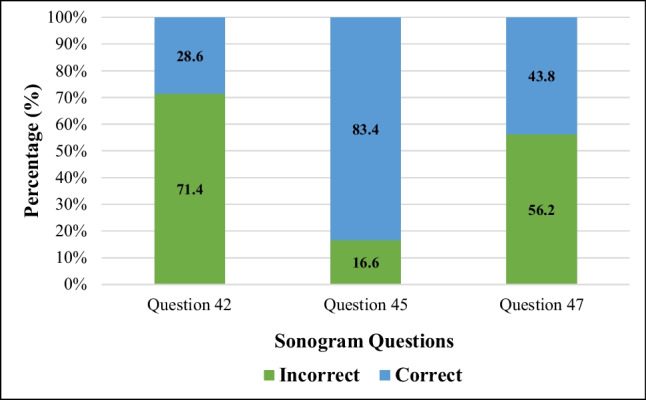
Stacked bar results of sonogram questions in practical test (*n* = 283). The results are graphed as percentage correct and incorrect answers per question

**Fig. 3:**
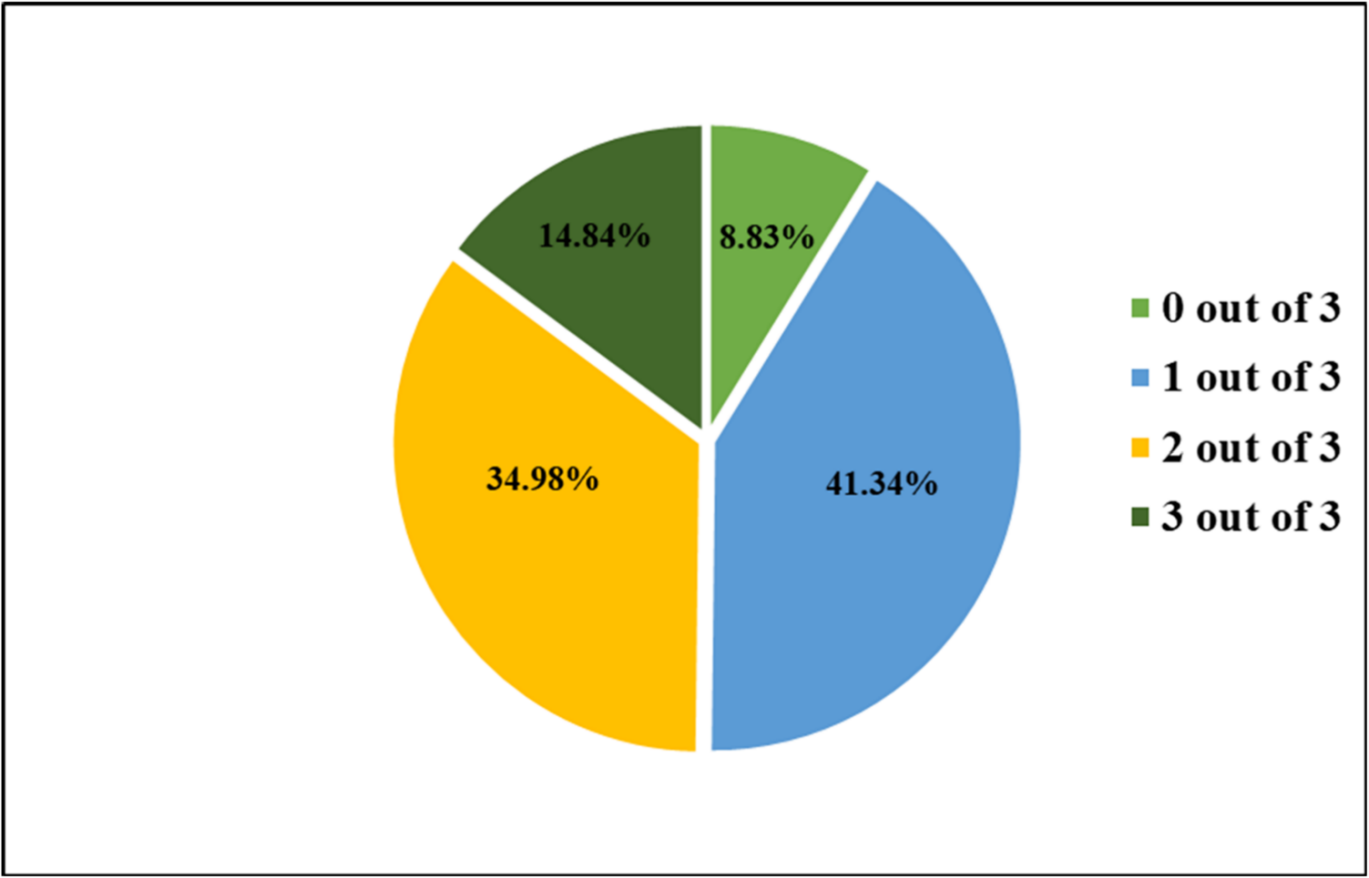
Pie chart of distribution of sonogram question test marks (*n* = 283). The results are graphed as percentage of the group that obtained 0/3, 1/3, 2/3, and 3/3 for the sonogram questions in the practical test

A summary of the results obtained from the questions is illustrated in Fig. 2. The lowest average was obtained in question 42 (28.6%) and the highest in question 45 (83.4%). The average for all the questions combined was 51.94%. Regarding the sonogram questions, 8.83% of the group was unable to answer any of the three questions correctly, whereas 14.84% answered all three questions correctly. A summary of the distribution of marks within the group is presented in Fig. 3.

### Qualitative Data

#### Participant Identifiers

The participants were identified by number, as well as the gender they identified with, as per the survey.

#### Generated Themes and Subthemes

Upon completion of the survey, students were granted the opportunity to describe how access to their own handheld, wireless ultrasound devices would aid in their preparation for the clinical realm, with specific regard to under-resourced areas (first open-ended question). They could add any additional commentary, specifically on their experience of the usage of ultrasound in teaching and learning anatomy (second open-ended question). Thirty-nine participants (95.12%) provided feedback on the first open-ended question, while 34 (82.93%) had complementary remarks to add. In total, 40 of the participants (97.56%) answered at least one of the two open-ended questions. Two themes, with four subthemes under theme one and three under theme two, were developed from the data analysis. Table 2 displays the categorized themes and subthemes.

**Table 2:** Themes and subthemes developed from the open-ended questions

Themes	Subthemes
1) **Instructional integration of ultrasound**	o Ultrasound competency o Setting o Means of instruction o Enhancement of anatomical comprehension
2) **Ultrasound within the clinical setting**	o Patient management o Hospital efficiency o Convenience

Theme One: Instructional Integration of Ultrasound.

This theme describes the introduction of ultrasound teaching within the educational realm and its accompanying factors.

### Ultrasound Competency

Competency in ultrasound refers to a person’s ability to efficiently use the ultrasound device. The participants identified both positive and negative aspects concerning their experience with ultrasound, during the practical sessions, in aiding their ultrasound skills. The participants commented on the benefits of exposing students to ultrasound in a practical manner, as it will aid them in gaining a better comprehension of ultrasound’s working principle, alongside the value of gaining pre-clinical knowledge and experience.

You need to have good pre-clinical knowledge and experience. A handheld wireless ultrasound device would greatly aid in this regard. P16M

The participants also made mention of the value of ultrasound training in improving their confidence with regard to usage of the device, as they would become more comfortable with its mechanisms, as well as interpretation of the ultrasonographic images, thus allowing for more self-study to occur pertaining to the latter.

It would help to build our confidence in performing and interpreting ultrasound images. P17F

However, many of the participants felt that, due to their limited exposure to and knowledge about ultrasound, they did not fully comprehend what they saw during the ultrasonic visualization, as they had no prior experience with it. Furthermore, they were not confident in their ability to use the device comfortably.

Was quite difficult for me to fully understand everything and where these structures are as this was my first clinical exposure to ultrasound. P6F

As a result, it was mentioned that more time is needed to become adequately prepared for ultrasound application within both the pre-clinical and clinical settings. Participants saw the value in continuous practice of ultrasound, with more sessions to develop their ultrasound skills and comprehension of ultrasound images.

Would’ve liked more practice with the handheld, wireless ultrasound device. I think there is a learning curve to this device. P10F

### Setting

Setting refers to the environment where students can learn ultrasound and improve or exchange their knowledge and experience thereof to each other. A few of the participants valued the benefit of using ultrasound within the pre-clinical setting, as they felt the necessary knowledge and skills should be obtained prior to application in the clinical realm and suggested that it should be incorporated during the practical sessions, with pre-preparation in a classroom setting.

To be able to apply your skills in the clinical setting, you need to have good pre-clinical knowledge. P16M

One participant, however, saw the integration of ultrasound training within the clinical realm as potentially more effective.

Being integrated more into our learning throughout our clinical training. P3F

### Means of Instruction

The third subtheme refers to the teaching methods and learning activities that are used to deliver the ultrasound curriculum in an educational setting. Most of the responses indicated that the practical ultrasound sessions should be better planned, with more thought going into the delivery and execution thereof.

Execution was very poor and quite disorganised. P1M

Participants also suggested that more time should be allocated to ultrasound use in the future, as they felt that the time spent on ultrasound scanning during the practical session was not enough for them to become comfortable with the device and confident in the structures they were seeing.

More exposure to ultrasound, with an info session on basics in ultrasound, would really help. P6F

Furthermore, participants mentioned the value of having an introductory session on ultrasound prior to the practical session, which will enable students to become familiar with the basic principles of ultrasound.

More detailed explanation of the usage of the device and interpretation of the results. P20M

### Enhancement of Anatomical Comprehension

This category involves notable progress in the development of anatomical knowledge and the capacity for fully understanding structural visualization. Participants felt that the use of ultrasound aided them in understanding anatomy and reinforced their knowledge of different anatomical structures, enabling them to discern the structures they were eliciting.

It has reinforced my understanding of anatomy. P41F

One participant mentioned that the use of ultrasound in viewing anatomical structures brought about a different perspective, as these structures viewed ultrasonically, in real time, differ significantly from those viewed in cadaveric specimens.

It brings about a different perspective. P23M

A few of the participants also saw the clinical application and benefit of having a comprehensive understanding of anatomical concepts, which can be applied to improve patient management within the clinical setting.

It helped greatly in imagining the same anatomical structures in both disease/injury-free patients and their healthy counterparts. P16M

#### Theme Two: Ultrasound Within the Clinical Setting

Theme two describes the utilization of ultrasound within the clinical realm, with the stated subthemes developing as key factors.

### Patient Management

Patient management describes the interaction between the healthcare team and a patient, which includes, but is not limited to communication, examination, evaluation, diagnosis, and prognosis. The participants mentioned that access to handheld, wireless ultrasound devices would enable them, as future clinicians, to be more focused on the management of their patients and their treatment, as well as improve their understanding of the patients they are treating.

Enable us to treat patients more efficiently. P17F

The responses also indicated that wireless ultrasound would aid significantly in visualizing a patient’s anatomy more comprehensively, thus aiding in clinical tasks requiring the application of anatomical knowledge.

During rounds when students have to draw blood, we sometimes struggle to feel the veins of the patients. This would help us visualize the veins better and understand our patients better. P15F

Furthermore, participants mentioned the value of having a device that allows quick and immediate ultrasound scanning, which could lead to faster, more accurate diagnostic outcomes and the identification of pathology.

Scan the patient immediately in the room without making them wait for future dates and issues with transport to the nearest facility. P25F

One participant highlighted the value of portable, handheld, wireless ultrasound scanning in the provision of medical services in areas with limited resources.

The best medical care to our patients in an under-resourced setting. P39F

### Hospital Efficiency

This category refers to the highest level of performance in a clinical setting in which the least inputs are required to achieve the greatest output. Most of the participants felt that handheld, wireless ultrasound would be extremely beneficial in a clinical setting, as it will contribute to the effectiveness of systemic operations within the clinical realm.

Would help greatly in aiding certain clinical associations. P35M

The participants also felt that less time will be wasted within the clinical setting when wireless ultrasound can be utilized and it will allow them to not be completely dependable on senior doctors and nurses, who are already busy with numerous tasks, to teach them the skills of ultrasound.

Allow us students to not be completely reliable on senior doctors and nurses who are already busy and understaffed to teach us. P10F

The participants also highlighted the potential value of wireless ultrasound scanning in under-resourced areas.

Provide access to ultrasound technology in areas where it wouldn’t otherwise be available. P33F

### Convenience

The third subtheme, convenience, describes the suitability for performing ultrasound or fulfilling the requirements necessary for scanning efficiently, without any difficulty. In comparison to bigger machines, the participants believed the device is more cost-effective, easier to handle, convenient, and portable as well as pocket friendly.

Ultrasound machines too big to fit between the beds of the patients, so this is much easier to manoeuvre. P32F

On-hand tool in your pocket to give you a wider viewpoint. P3F

One participant specifically mentioned that the handheld and wireless components of ultrasound devices have the potential to improve access to ultrasound imaging modalities.

Handheld and wireless the accessibility to this resource will be increased. P22M

## Discussion

Several previous studies have integrated ultrasound sessions into anatomy courses and found numerous benefits [2, 4, 18, 24, 27], but the current study is unique in its usage of portable, handheld, wireless ultrasound devices. Overall, the participants’ feedback was positive and indicated both an enjoyable and useful experience with the ultrasound devices during the MSK practical session. This resonates with the findings of Correia et al. [4], where students expressed their excitement about the novelty of using ultrasound during an anatomy practical session. Additionally, students appreciated the opportunity to see living anatomy to augment anatomy cadaveric laboratory learning [4]. The utilization of ultrasound allows students to view anatomical structures in real time, while observing physiological processes, e.g., the flow of blood. As a result, a new perspective is gained, that is quite distinctive from the structural characteristics of cadaveric specimens.

The participants also commented on ultrasound scanning reinforcing their understanding of anatomical structures. Evidence in the literature supports participants’ feedback that ultrasound enhanced learning and comprehension of living anatomy [4, 17, 29, 33]. Identifying organs and structures in the living human body, with the help of ultrasound, aided in strengthening students’ previous anatomy knowledge gained from other sources, while practically demonstrating anatomy concepts. Ultrasound has been viewed by the students as an innovative, exciting, and engaging approach to enhancing their reasoning skills while learning clinical anatomy, which aligns with the findings of Brown et al. [54] and Swamy and Searle [5]. The study of anatomy involves exploring and understanding three-dimensional (3D) anatomical structures, making it an extremely visual, high-content, and practical course [4]. Medical education has traditionally utilized cadavers to teach anatomy and has been considered an integral part of the course [5]. In surgical specialties, radiology, internal medicine, dentistry, and other allied health professions, students were less satisfied with the sessions when they had difficulty recognizing the anatomy, unaccustomed to the ultrasound scanning format, and could not understand the scan and probe orientation. This is in accordance with the findings of this study, where the low student agreement observed in question 5 indicates that the in-person introductory explanation of the fundamentals of ultrasound was not adequate before the hands-on practical session. The participants made mention of the lack of organization within the ultrasound practical session, highlighting the insufficient amount of time allocated to the proper introduction to ultrasound principles, as their lack of previous exposure to and knowledge of ultrasound imaging modalities resulted in incomprehension of ultrasonographic visualized anatomical structures. Even though most of the participants valued ultrasound demonstration as a necessary add-on to traditional dissection sessions during the anatomy practical session, the integrated session was experienced as rushed and overwhelming alongside achieving their outcomes for the practical dissection session. A possible explanation might be that students were unfamiliar with the dissection hall and its practical experience, as they had no practical sessions prior to this first (and only) MSK practical component, due to restrictions imposed by COVID-19 from March 2020.

Participants thus suggested that more time be dedicated to ultrasound use in the future, as they felt that the time spent on ultrasound scanning during the practical session was insufficient to become comfortable with the device and confident in the structures visualized. The results of this study align with that of other researchers that conducted similar sessions with ultrasound, where students indicated that they would like to attend more ultrasound sessions and have more time and opportunities to have hands-on practical experience with the ultrasound device [2, 4, 17].

Contrary to a study done by Chen et al. [49], which did not include the evaluation of ultrasound knowledge and ultrasound imaging, specifically to keep the emphasis on the efficacy to study anatomy itself, this study integrated ultrasound assessment employing sonogram questions in the routine practical test. The students obtained a low average for the ultrasonographic Sect. (51.94%), indicating that they are not confident in their ability to identify anatomical structures on ultrasound images. These average results were verified as question 8 obtained the lowest value of agreement among the students, which inquired if students were confident in their capability to generate an image of an organ or other structures in the human body using a handheld, wireless ultrasound probe. The result may also be attributed to the vast difference between ultrasound images and gross anatomy in a cadaver, as organs and structures in cadavers appear quite different from monochromatic images [49]. Even though the students had the opportunity to view these structures with the aid of ultrasound imaging, they only had one hands-on ultrasound training session, which did not aid them sufficiently in interpreting ultrasound images and applying their anatomical knowledge correctly, as discussed earlier with students’ desires for more ultrasound sessions. These findings differ from the results obtained by Kefala-Karli et al. [2], who observed that, because of hands-on ultrasound training during the gross-anatomy course, students were able to interpret ultrasound images quicker which may be associated with a faster retrieval of anatomy knowledge required to analyze images and discern patterns.

Moreover, research conducted by Frankhauser et al. [14] showed that providing each student with their own ultra-portable scanner, which they can use both on their own time and in the presence of an instructor, might be a more beneficial option for achieving pre-clinical ultrasound outcomes. This resonates with the results in this study, where participants expressed the desire to practice ultrasound scanning in both the pre-clinical and clinical setting, per their schedules and indicated that their longitudinal learning experience at Stellenbosch University would be enhanced if they had access to wireless ultrasound devices. The usefulness of implementing ultrasound training, with devices readily available, in the undergraduate medical curriculum within the framework of longitudinal learning processes, emerged in the commentary. This is complementary to the observations of Ireson et al. [16].

Students’ desire for additional ultrasound instruction also developed as a subtheme from qualitative analysis, specifically in relation to more image acquisition instruction, exposure to additional ultrasound techniques, and practice with ultrasound devices, to develop ultrasound competency, as this is required for successful clinical application of ultrasonography. As image acquisition and diagnostic skills are developed with practice, exposure to more live ultrasound demonstrations and acquiring radiology interpretation skills in a pre-clinical setting, e.g., anatomical practical sessions, would adequately prepare future clinicians for efficient POCUS application in the clinical realm. Students expressed interest in expanding ultrasound skills and gaining hands-on experience, with focused scanning, to apply their anatomical knowledge in the clinical setting, where it is ultimately utilized. This reflects the observations from Sa’hari Ramli et al. [8], indicating that assisting students to construct a connection between theory and clinical practice would be made easier if radiological images were incorporated into anatomy lessons. Thus, students learn the importance of grasping anatomy knowledge and retaining it for future career application [8]. As mentioned by participants in this study, it is beneficial to have an in-depth comprehension of various anatomical concepts, particularly for applicability in clinical practice, as this can contribute to the improvement of patient care.

Most of the participants found the handheld wireless ultrasonography probe convenient for practice, as observed in the high student agreement in question 6. This is confirmed in the additional commentary, where convenience developed as one of the subthemes. The participants appreciated the practicality of the device, especially for prospective use in the clinical realm, and made special mention of its accessibility, promptness, and the benefit of having a readily available imaging modality on hand. The results presented here agree with those of Ireson and colleagues, where students found that handheld ultrasound devices were easy to use and led to an improved understanding of 3D anatomy [16].

Many of the participants agreed that the healthcare system and patient outcomes, especially in under-resourced areas, will be enhanced if each medical student had their own wireless ultrasound device (question 13). These results were also verified by subthemes developed from the qualitative analysis, as commentary showed that the management, comprehension, and treatment of patients, by prospective clinicians, will be enabled by easy access to handheld, wireless ultrasound devices. Feedback revealed that the latter will also aid in various clinical assignments and enable unrestricted scanning, a feature that is extremely useful during an emergency, which is also mirrored in the literature [10, 12, 19].

Some participants also highlighted the amount of time that can be saved within the clinical realm with wireless ultrasound utilization, due to its convenience and ready availability, as previously mentioned. Furthermore, by implementing the use of wireless ultrasound, future clinicians will be less dependent on senior medical doctors and nurses to provide them with ultrasound training, as these colleagues are working on a high volume of medical projects and assignments with limited personnel. This is in keeping with a study done by Lunn-Collier et al. [11], where it was found that establishments staggering under staffing shortages could be aided by the incorporation of innovation in technology, e.g., handheld, wireless ultrasound. The potential value of such additions in the healthcare system of under-resourced areas specifically was noteworthy.

Furthermore, comments by the participants referenced the assistance that access to portable, handheld wireless ultrasound scanning would bring to clinical facilities and the significant contribution it would make to providing healthcare to the public in these areas. Access to healthcare is limited in under-resourced areas, due to several factors [31, 42, 44] which include but is not limited to indigence, lack of resources, concerns and misconceptions of individuals, and the instability of the electricity supply in countries such as South Africa. The findings in this study align with those of Naganuma and Ishida [39], who revealed that wireless, handheld ultrasound scanning enables transmitting ultrasound data to rural clinical settings, thus providing inexperienced sonographers with ultrasound images to aid in the improvement of patient care. Consequently, exposure to handheld, wireless ultrasound scanning enables future clinicians to overcome the limitation concerning imaging modalities in under-resourced medical facilities [14, 30].

The study is limited in the sense that it was only conducted at one institutional setting, namely Stellenbosch University, allowing the results to be generalized only to a certain extent. Outcomes may vary in different organizational or didactical settings. The study population only included third-year medical students, which limited the sample size. The study was also restricted to visualizing the MSK system, specifically the vasculature of the upper limb, with ultrasound, and it would be noteworthy to investigate if students’ perceptions of the use of handheld, wireless ultrasound devices to learn anatomy would be influenced differently if more anatomical regions could be explored with ultrasound. Another restriction was the limited time available for students to use the ultrasound devices during the practical session. In this study, evaluation of long-term retention of ultrasonographic skills and principles was not done and it would be beneficial to investigate the long-term benefits of pre-clinical exposure to ultrasonography and clinical practice.

Considering the findings of this study, future studies could focus on other health professions students regarding their perspectives on the use of handheld, wireless ultrasound devices as a learning tool for anatomy, with specific clinical application to their future profession. Perceptions could be gathered before using ultrasound during the practical sessions, as well as afterwards, to see if there is a significant change in perceptions after ultrasound exposure. A follow-up on the cohort of medical students that participated in this specific study, over the course of their clinical training, would also be insightful. The latter could determine if they perceived the incorporation of ultrasound during their practical anatomy sessions improved their preparations for the clinical realm. Moreover, the perceptions of anatomy educators could also be explored regarding the educational integration of ultrasound, alongside a needs assessment pertaining to the latter, and a standardized curriculum for the implementation of ultrasound within the anatomy curriculum could be designed, should the needs assessment indicate a requirement thereof.

## Conclusion

This study aimed to explore undergraduate medical students’ perceptions of the use of handheld wireless ultrasound scanning to enhance knowledge and understanding of anatomy for clinical application. The results of this study indicate that participants view ultrasound as an essential auxiliary to practical anatomy dissection sessions and their longitudinal learning experience would be enhanced with access to a wireless ultrasound device. Moreover, the participants had a desire to engage in structured longitudinal learning through ultrasound inclusion in the curriculum, as it would aid them in ultrasound application in the clinical realm. The limited time allocated to ultrasound exposure within the practical session, as well as the effectiveness of the in-person introductory explanation of the fundamentals of ultrasonography, was the most notable drawbacks.

Appreciation was shown for the convenience, portability, and ready availability of handheld wireless ultrasound scanning, with the potential to streamline various processes in the clinical setting, alongside improving patient outcomes and hospital efficiency. The implementation of handheld wireless ultrasonography could ease dependency on senior clinicians and nursing staff for teaching fundamental ultrasound skills to future medical practitioners, enabling them to become comfortable with ultrasonography through continual self-study. This enhances future clinicians’ abilities to overcome the challenges and limitations under-resourced areas are experiencing with imaging modalities, thus enabling them to improve the healthcare system and provide access to diagnostic procedures in these resource-constrained areas.

The material in this manuscript was presented in a poster format at Stellenbosch University’s 66th Annual Academic Day (August 2022) as well as at the South African Association of Health Educationalists Conference (June 2023).

## Data Availability

The anonymized data supporting the findings of this study are available upon reasonable request from the corresponding author.
